# A multicopy suppressor screening approach as a means to identify antibiotic resistance determinant candidates in *Yersinia pestis*

**DOI:** 10.1186/1471-2180-8-122

**Published:** 2008-07-21

**Authors:** Karen L Stirrett, Julian A Ferreras, Sebastian M Rossi, Richard L Moy, Fabio V Fonseca, Luis EN Quadri

**Affiliations:** 1 Department of Microbiology and Immunology, Weill Medical College of Cornell University, 1300 York Avenue, New York, New York 10021, USA; 2 Universidad Nacional de Misiones, Facultad de Ciencias Exactas, Quimicas y Naturales, Felix de Azara 1552, C.P. N3300LQH, Posadas, Argentina; 3 Medical College of Georgia, Vascular Biology Center, 1459 Laney Walker Boulevard, CB 3201B, Augusta, Georgia 30912, USA

## Abstract

**Background:**

*Yersinia pestis *is the causative agent of plague and a potential agent of bioterrorism and biowarfare. The plague biothreat and the emergence of multidrug-resistant plague underscore the need to increase our understanding of the intrinsic potential of *Y. pestis *for developing antimicrobial resistance and to anticipate the mechanisms of resistance that may emerge in *Y. pestis*. Identification of *Y. pestis *genes that, when overexpressed, are capable of reducing antibiotic susceptibility is a useful strategy to expose genes that this pathogen may rely upon to evolve antibiotic resistance via a vertical modality. In this study, we explored the use of a multicopy suppressor, *Escherichia coli *host-based screening approach as a means to expose antibiotic resistance determinant candidates in *Y. pestis*.

**Results:**

We constructed a multicopy plasmid-based, *Y. pestis *genome-wide expression library of nearly 16,000 clones in *E. coli *and screened the library for suppressors of the antimicrobial activity of ofloxacin, a fluoroquinolone antibiotic. The screen permitted the identification of a transcriptional regulator-encoding gene (*robA*_Yp_) that increased the MIC_99 _of ofloxacin by 23-fold when overexpressed from a multicopy plasmid in *Y. pestis*. Additionally, we found that *robA*_Yp _overexpression in *Y. pestis *conferred low-level resistance to many other antibiotics and increased organic solvent tolerance. Overexpression of *robA*_Yp _also upregulated the expression of several efflux pumps in *Y. pestis*.

**Conclusion:**

Our study provides proof of principle for the use of multicopy suppressor screening based on the tractable and easy-to-manipulate *E. coli *host as a means to identify antibiotic resistance determinant candidates of *Y. pestis*.

## Background

*Yersinia pestis *(*Yp*) is one of the most virulent known bacteria [1] and a potential agent of bioterrorism and biowarfare [2,3] included in the Category A of biological agents for public health preparedness against bioterrorism [4]. *Yp *is the etiologic agent of plague, a disease responsible for millions of human deaths during the history of civilization [5,6]. Cases are reported every year in many parts of the world [7] and the increasing number of worldwide cases has placed plague in the category of re-emerging diseases [8].

Patients with plague need prompt antibiotic treatment or else death may be unavoidable. The aminoglycosides streptomycin (STR) and gentamicin (GEN) are the preferred antibiotics for treatment, but a number of other drugs are also effective [9,10]. Tetracyclines [such as doxycycline (DOX)], chloramphenicol (CHL), or selected sulfonamides are the recommended antibiotics for prophylactic therapy in the event of exposure or high risk of exposure to *Yp *[2,9,10]. Fluoroquinolones have also been suggested for treatment and prophylaxis and are noted as a chemotherapeutic alternative against strains resistant to the first line anti-plague drugs [2,10].

The threat of bioterrorism-generated plague outbreaks with engineered (multi)drug-resistant *Yp *strains [2,3] and the documented outbreak of multidrug-resistant plague [11] underscore the need to develop alternative chemotherapeutic solutions to this disease. In line with this view, we are exploring the development of anti-infectives that target the high-affinity iron acquisition system of *Yp *[12-14] and may offer novel therapeutic possibilities [15]. The plague biothreat also underscores the need to increase our understanding of the intrinsic potential of *Yp *for developing antimicrobial resistance and to anticipate the mechanisms of resistance that may emerge in *Yp *clinical isolates in the future. With this consideration in mind, we explored herein the use of a multicopy suppressor screening approach as a means to expose antibiotic resistance determinant candidates in *Yp*. Multicopy suppressor screening has been useful to study potential drug targets or mechanisms of antibiotic resistance in other species [16]. We constructed a multicopy plasmid-based, *Yp *genome-wide expression library of nearly 15,000 clones in *E. coli *(*Ec*), a tractable and easy-to-manipulate surrogate bacterial host, and screened the library for suppressors of the antimicrobial activity of the fluoroquinolone antibiotic ofloxacin (OFX). Noteworthy, fluoroquinolones have been suggested by the Working Group on Civilian Biodefense as alternative drugs in the event of the use of aerosolized *Yp *as a bioweapon against a civilian population [2]. The screen permitted the identification of a gene that reduced the susceptibility of *Yp *to fluoroquinolones and other antibiotic classes when overexpressed from a multicopy plasmid. Our study provides proof of principle for the utilization of multicopy suppressor screening using an *Ec *host as a means to identify antibiotic resistance determinant candidates in *Yp*.

## Results and Discussion

### A multicopy suppressor screen led to the isolation of a *Y. pestis *genomic fragment involved in ofloxacin resistance

**Figure 1:**
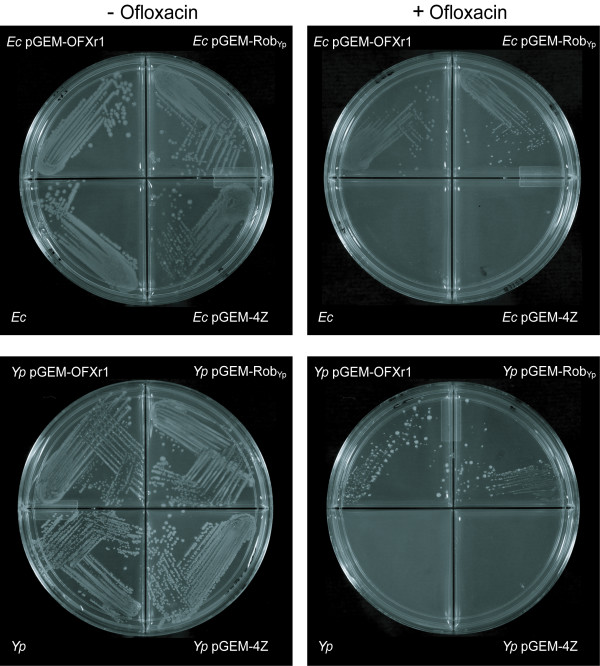
**Reduction of ofloxacin susceptibility conferred by plasmids pGEM-OFXr1 and pGEM-Rob_Yp_**. *E. coli *(*Ec*) and *Y. pestis *(*Yp*) strains were streaked on solid media without or with ofloxacin: 0.35 μg/ml (the concentration used in the screen) for *E. coli *and 0.15 μg/ml for *Y. pestis*. Ampicillin (100 μg/ml) was also added to the media for plasmid-carrying strains.

**Figure 2:**
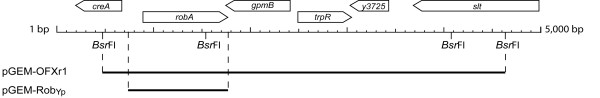
Genetic map of the *robA*_Yp_-containing region of the *Y. pestis *KIM chromosome and inserts of pGEM-OFXr1 and pGEM-Rob_Yp_.

We constructed a plasmid-based expression library of the *Yp *genome comprised of 15,648 *Ec *clones and screened the library for strains with reduced OFX susceptibility. A strain (*Ec *pGEM-OFXr1) selected in the screen exhibiting reduced susceptibility that was confirmed to be plasmid-mediated and transferable to *Yp *was chosen for further characterization (Figure 1). The plasmid (pGEM-OFXr1) carried by this strain was isolated and the restriction digestion pattern and sequence of its genomic insert were examined. This analysis revealed a 4,158-bp fragment (*Yp *KIM chromosome coordinates 4,137,482 to 4,141,639) (Figure 2). The 5' and 3' ends of the fragment included the 5' end of *y3722 *(*creA*) and the 3' end of *y3727 *(*slt*), respectively. The products of *creA *and *slt *are annotated as a conserved hypothetical protein and a putative soluble lytic murein transglycosylase, respectively, in the *Yp *genome database. The center of the fragment encompassed four genes: *y3723 *(*robA*, herein referred to as *robA*_Yp_); *y3724 *(*gpmB*); *y3725*; and *y3726 *(*trpR*). The products of *gpmB *and *trpR *are annotated as a putative phosphoglyceromutase and a putative regulator of tryptophan metabolism genes, respectively. The product of *y3725 *is annotated as a conserved hypothetical protein. Our *in silico *search for conserved domains (via CD-Search; please see Availability & requirements for more details) revealed the presence of an NTPase (PRK05074) domain in this protein. The NTPase domain is characteristic of proteins with pyrophosphatase activity [17,18]. This suggested that *y3725 *may be involved in nucleoside triphosphate metabolism. Lastly, the predicted product of *robA*_Yp _(RobA_Yp_) is annotated as an orthologue of *Ec *RobA (RobA_Ec_), a transcriptional regulator of unclear physiological function and member of the AraC/XylS family [19]. Importantly, overexpression of *robA*_Ec _and *Enterobacter cloacae robA *confers low-level resistance in *Ec *and *E. cloacae*, respectively, to a number of unrelated antibiotics [20-22]. Thus, the analysis of the insert in pGEM-OFXr1 suggested that *robA*_Yp _is responsible for the reduced OFX susceptibility observed in *Ec *pGEM-OFXr1 and *Yp *pGEM-OFXr1 (Figure 1). These results validate the utility of our library and suppressor screen approach as a means to identify antibiotic resistance determinant candidates in *Yp*.

### Overexpression of *robA*_Yp _affects susceptibility to multiple antibiotics

We investigated whether overexpression of *robA*_Yp _alone would reduce OFX susceptibility in *Ec *and, more importantly, in *Yp*. To this end, we evaluated the antibiotic susceptibility of *Ec *pGEM-Rob_Yp _and *Yp *pGEM-Rob_Yp_. These test strains carried pGEM-Rob_Yp_, a plasmid constructed by inserting the fragment encompassing *robA*_Yp _and its promoter region (identified by using the *robA*_Ec _promoter as reference [23]) into the vector pGEM-4Z. The antibiotic susceptibilities of these test strains were compared to that of the corresponding *Ec *pGEM-4Z and *Yp *pGEM-4Z control strains. These control and test strains were isogenic, except for the lack of the plasmid-borne *robA*_Yp_, and their growth in ampicillin (AMP)-containing liquid media was indistinguishable from that of their cognate test strains (not shown). A first examination of *Ec *pGEM-Rob_Yp _and *Yp *pGEM-Rob_Yp _indicated that these strains retained the reduced OFX susceptibility phenotype seen in *Ec *pGEM-OFXr1 and *Yp *pGEM-OFXr1 on solid media (Figure 1), thus indicating that *robA*_Yp _alone was sufficient to reduce OFX susceptibility. In view of this, we conducted further OFX susceptibility testing in liquid media. In addition, we compared the susceptibility of the test and control strains to two other fluoroquinolones [ciprofloxacin (CIP) and levofloxacin (LVX)], a quinolone (NAL), and antibiotics of other classes, including two tetracyclines [tetracycline (TET) and DOX], four aminoglycosides [STR, GEN, kanamycin (KAN), and apramycin (APR)], and CHL.

The IC_50 _and MIC_99 _values determined for the aforementioned antibiotics are shown in Table 1. Comparison of the OFX IC_50 _and OFX MIC_99 _values of the test strains and their respective control strains revealed that overexpression of *robA*_Yp _reduced OFX susceptibility in both *Yp *and *Ec*. In *Yp*, *robA*_Yp _overexpression increased OFX IC_50 _and OFX MIC_99 _values by 5-fold and 23-fold, respectively. The IC_50 _and MIC_99 _values of CIP, LVX, and NAL also increased significantly (3- to 5-fold change range) in *Yp *pGEM-Rob_Yp _compared with *Yp *pGEM-4Z. The reduced OFX and LVX susceptibility of *Yp *pGEM-Rob_Yp _was also revealed by time-kill experiments described below. In *Ec*, *robA*_Yp _overexpression produced an increase in the IC_50 _and MIC_99 _of the fluoroquinolone antibiotics (2- to 4-fold change range), but had no significant effect (<2-fold change) on NAL susceptibility.

**Table 1:** Effect of *robA*_Yp _overexpression on antibiotic susceptibility

	IC_50 _(μg/ml)^a^			FC^c^	MIC_99 _(μg/ml)^b^			FC
				
*Y. pestis*	no plasmid	pGEM-4Z	pGEM-Rob_Yp_		no plasmid	pGEM-4Z	pGEM-Rob_Yp_	
Ofloxacin	0.01	0.01	0.05	5	0.03	0.03	0.7	23
Ciprofloxacin	0.009	0.009	0.04	4	0.02	0.02	0.09	5
Levofloxacin	0.01	0.01	0.04	4	0.02	0.02	0.09	5
Nalidixic Acid	0.5	0.8	2	3	2	2	6	3
Chloramphenicol	0.3	0.4	0.9	2	1	1	3	3
Tetracycline	0.7	1	6	6	1	3	10	3
Doxycycline	0.4	0.5	1	2	0.6	1	3	3
Kanamycin	1	0.9	0.4	0.4	2	2	2	1
Apramycin	2	3	1	0.3	6	6	4	0.7
Streptomycin	1	2	0.8	0.4	3	3	3	1
Gentamicin	0.4	0.4	0.2	0.5	1	0.4	0.4	1
				
*E. coli*	no plasmid	pGEM-4Z	pGEM-Rob_Yp_		no plasmid	pGEM-4Z	pGEM-Rob_Yp_	

Ofloxacin	0.03	0.04	0.09	2	0.08	0.2	0.4	2
Ciprofloxacin	0.01	0.009	0.04	4	0.05	0.08	0.2	3
Levofloxacin	0.04	0.04	0.1	3	0.2	0.2	0.4	2
Nalidixic Acid	27	26	28	1	83	100	100	1
Chloramphenicol	0.3	0.3	0.8	3	2	2	13	7
Tetracycline	0.8	0.6	2	3	3	3	10	3
Doxycycline	0.3	0.2	0.6	3	1	0.6	3	5
Kanamycin	2	1	2	2	10	6	6	1
Apramycin	3	3	4	1	21	17	21	1
Streptomycin	3	2	3	2	17	13	13	1
Gentamicin	0.9	0.6	2	3	6	5	8	2

^a ^IC_50 _values were calculated from sigmoidal curves fitted to triplicate sets of dose-response data. ^b ^MIC_99 _values are means of triplicates. ^c ^Fold change (FC) values were calculated as the ratio of the IC_50 _or MIC_99 _of the pGEM-Rob_Yp _transformants to the IC_50 _or MIC_99 _of the pGEM-4Z transformants. IC_50_, MIC_99_, and FC values <1 and values >1 were rounded to one significant digit and to the nearest non-fractional number, respectively.

In both *Yp *and *Ec*, *robA*_Yp _overexpression also correlated with an increase in the IC_50 _and MIC_99 _of the two tetracyclines tested and CHL (2- to 7-fold change range). No substantial impact (<2-fold change) on the MIC_99 _values of four aminoglycosides tested was detected in *Yp *upon overexpression of *robA*_Yp_. Interestingly, however, the IC_50 _values of these aminoglycosides were reproducibly and consistently lower (2- to 3-fold reduction range) in *Yp *pGEM-Rob_Yp _compared with *Yp *pGEM-4Z. These results indicated that *robA*_Yp _overexpression increased the susceptibility of *Yp *to aminoglycosides. The hypersensitivity of *Yp *pGEM-Rob_Yp _to aminoglycosides was also observed in time-kill experiments described below. Aminoglycoside hypersensitivity was not observed in *Ec *pGEM-Rob_Yp_. On the contrary, the strain had a modest decrease in the susceptibility to GEN, STR, and KAN (2- to 3-fold change range) relative to *Ec *pGEM-4Z.

Overall, the phenotypic comparison of the antibiotic susceptibility of pGEM-Rob_Yp_*-*bearing strains and pGEM-4Z-bearing strains clearly demonstrates that *robA*_Yp _overexpression affects antibiotic susceptibility in both *Yp *and *Ec*, yet in a noticeably species-specific manner. As discussed below, the effects on antibiotic susceptibility induced by *robA*_Yp _overexpression are likely due to an upregulation of efflux pumps. Thus, the species-specific differences in antibiotic susceptibility are probably produced by species-specific differences in efflux pump upregulation.

### Overexpression of *robA*_Yp _in *Y. pestis *reduces killing by fluoroquinolones but enhances killing by aminoglycosides

**Figure 3:**
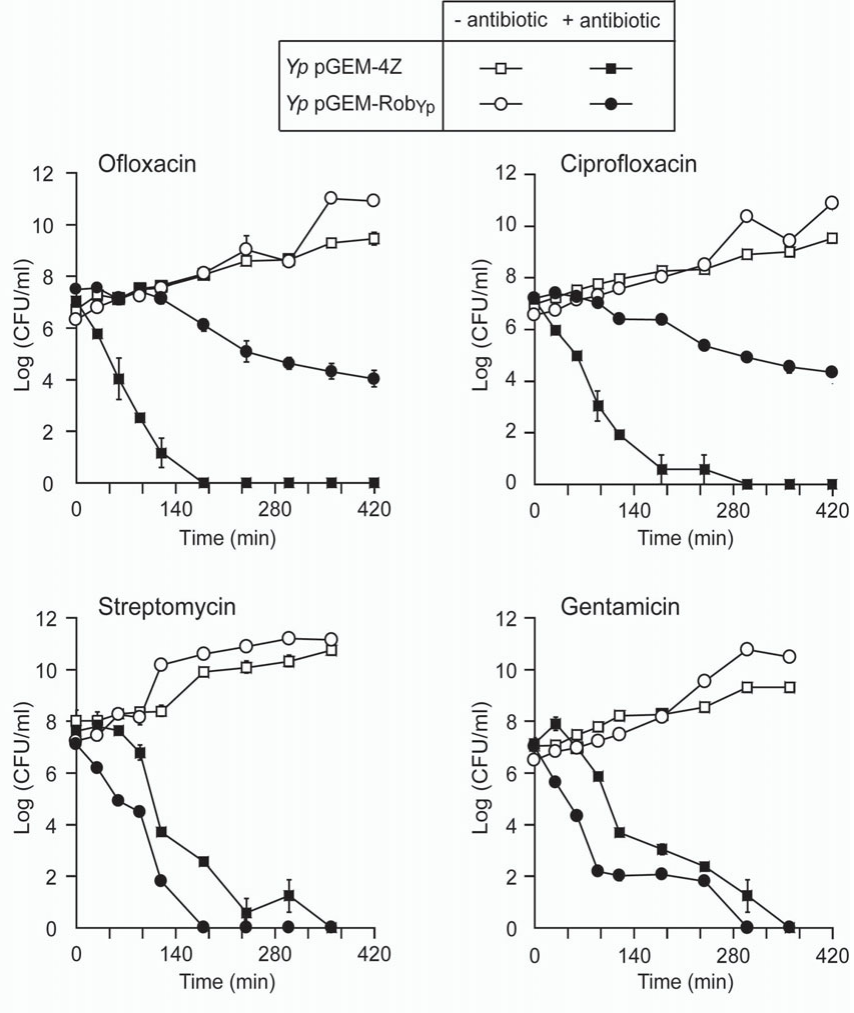
**Effect of *robA*_Yp _overexpression on the rate of *Y. pestis *killing by fluoroquinolones and aminoglycosides**. *Yp *pGEM-Rob_Yp _(overexpressing *robA*_Yp_) and *Yp *pGEM-4Z (vector control) were treated with the indicated antibiotics at 5 × MIC_99_. The means of triplicate treated cultures were plotted and standard error bars are shown.

The comparative analysis of IC_50 _and MIC_99 _values described above indicated that *Yp *pGEM-Rob_Yp _has reduced fluoroquinolone susceptibility and increased aminoglycoside susceptibility compared with *Yp *pGEM-4Z. To further probe these phenotypes, we examined the killing kinetics of these two strains when exposed to OFX, CIP, STR, and GEN (Figure 3). The profiles of the time-kill curves for OFX and CIP demonstrated that the *Yp *pGEM-4Z control was more rapidly killed by the fluoroquinolones than *Yp *pGEM-Rob_Yp_. Conversely, the profiles of the time-kill curves for STR and GEN revealed that *Yp *pGEM-Rob_Yp _was more rapidly killed by the aminoglycosides than the *Yp *pGEM-4Z control. Both strains had comparable growth in the absence of fluoroquinolone or aminoglycoside antibiotics during the time frame of the time-kill assays. The contrasting effects of *robA*_Yp _overexpression on fluoroquinolone- and aminoglycoside-mediated killing are consistent with the results of the comparative analysis of IC_50 _and MIC_99 _values (Table 1). The observed aminoglycoside hypersensitivity is somewhat unexpected and contrasts with both the reduced susceptibility observed for all other antibiotics tested and the increased tolerance to organic solvents described below.

### Overexpression of *robA*_Yp _increases tolerance to organic solvents

**Figure 4:**
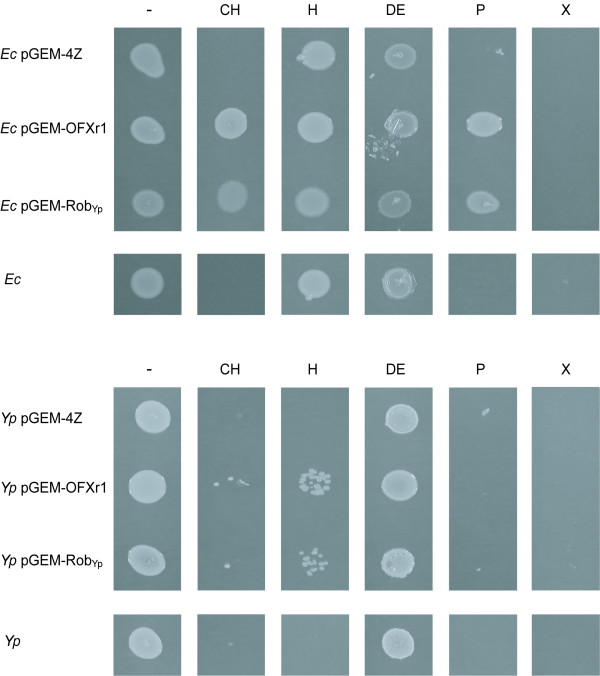
**Effect of *robA*_Yp _overexpression on organic solvent tolerance**. Cultures of *Y. pestis *(*Yp*) and *E. coli *(*Ec*) strains carrying the plasmids indicated were spotted on solid medium. The surface of the medium was overlaid with the organic solvent and growth was recorded after incubation. H, *n*-hexane; CH, cyclohexane; P, *n*-pentane; X, *p*-xylene; DE, diphenyl ether; -, no solvent control.

We investigated whether *robA*_Yp _overexpression affected the susceptibility of *Yp *and *Ec *to O_2_^.-^-generating compounds (paraquat, menadione, and plumbagin), heavy metals (zinc, cobalt, and copper), and organic solvents (*n*-pentane, *n*-hexane, cyclohexane, *p*-xylene, and diphenyl ether). No effect on the susceptibility to O_2_^.-^-generating compounds, cobalt, and copper was observed (not shown). Conversely, overexpression of *robA*_Yp _drastically increased organic solvent tolerance in both *Yp *and *Ec *(Figure 4) and reduced the susceptibility of *Yp *to zinc (not shown). Overexpression of *robA*_Yp _increased the tolerance of *Yp *to *n*-hexane and cyclohexane and the tolerance of *Ec *to cyclohexane and *n*-pentane. All the *Yp *and *Ec *strains were resistant to diphenyl ether and sensitive to *p*-xylene. *Ec *was also resistant to *n*-hexane, a result that is in agreement with previous reports [24]. These findings parallel the reduction of organic solvent susceptibility induced by *robA*_Ec _overexpression in *Ec *[21].

### Overexpression of *robA*_Yp _in *Y. pestis *induces changes in efflux pump gene expression

In *Ec*, the RobA_Ec_-induced multidrug resistance and solvent tolerance phenotypes have been shown to be largely dependent on the upregulation of the multidrug efflux pump AcrAB, which belongs to the resistance-nodulation-cell division (RND) superfamily [25,26]. With this precedent in mind, we compared the expression of genes belonging to 34 drug efflux pumps between *Yp *pGEM-Rob_Yp _and *Yp *pGEM-4Z using quantitative real-time PCR (qRT-PCR). These pumps were identified using a variety of bioinformatic approaches (see Methods) to compile an extensive list that included most, if not all, putative drug efflux pump systems encoded in the genome of *Yp*. It is worth mentioning as a reference that there are 37 drug efflux pumps annotated in the *Ec *genome [27,28]. Our expression analysis detected transcripts for 33 of the 34 genes investigated and revealed that four efflux pumps were significantly upregulated (≥ 5-fold change) in *Yp *pGEM-Rob_Yp _compared with *Yp *pGEM-4Z (Table 2). Interestingly, two of these upregulated pumps (*y3392-y3393 *and *y1050-y1049*) are *Ec *AcrAB homologs. The other two upregulated pumps (*y2173 *and *y0010*) belong to the major facilitator superfamily (MFS). The transcript level of *hasF *(*y3516*), encoding the ortholog of *Ec *TolC, which is the outer membrane protein channel that partners with *Ec *AcrAB and other RND and MFS pumps [29,30], was drastically upregulated as well (5.8-fold change; not shown).

**Table 2:** Effect of *robA*_Yp _overexpression on the transcript levels of efflux pump genes in *Y. pestis*

Gene name^a^	University of Wisconsin^b^	TIGR	SANGER	Pump family or protein function^c^	FC^d^
(*floR*)	*y2173*	NT02YP2579	YPO2148	MFS	**12.7 ± 2.5**
(*yieO*)	*y0010*	NT02YP0010	YPO0009	MFS	**8.6 ± 1.1**
(acrA*-acrB)	y3392*-y3393	NT02YP4041	YPO1000	RND	**6.9 ± 0.4**
acrA*-acrB	y1050*-y1049	NT02YP1227	YPO3132	RND	**5.0 ± 0.4**
(acrA*-acrB)	y3760*-y3759	NT02YP4463	YPO0420	RND	2.7 ± 0.1
*-*	*y4041*	NT02YP4789	YPO4020	DMT	1.7 ± 0.9
yegM*-yegN-yegO-yegB	y1386*-y1385-y1384-y1383	NT02YP1646	YPO2847	RND	1.4 ± 0.5
(ybjY*-ybjZ)	y2814*-y2813	NT02YP3366	YPO1364	ABC	1.4 ± 0.4
(acrA*-acrB)	y0702*-y0703	NT02YP0804	YPO3483	RND	1.3 ± 0.3
emrA*-emrB	y0922*-y0921	NT02YP1066	YPO3267	MFS	1.3 ± 0.4
(macA*-macB)	y1481*-y1480	NT02YP1756	YPO2999	RND	1.3 ± 0.5
*ygeD*	*y3180*	NT02YP3786	YPO0792	MFS	1.3 ± 0.2
*fieF*	*y0060*	NT02YP0067	YPO0077	CDF	1.2 ± 0.1
(*abgT*)	*y3402*	NT02YP4052	YPO1008	IT	1.1 ± 0.2
*acrD*	*y1439*	NT02YP1705	YPO3043	RND	1.1 ± 0.1
*sugE*	*y0613*	NT02YP0702	YPO0355	SMR	1.1 ± 0.4
(*ynfM*)	*y2108*	NT02YP2497	YPO2266	MFS	1.1 ± 0.4
*bcr*	*y2916*	NT02YP3488	YPO1267	MFS	1.1 ± 0.3
aaeA*-aaeB	y0178*-y0177	NT02YP0192	YPO3685	ArAE	1.1 ± 0.0
(yjcR*-Q)	y3558*-y3559	NT02YP4231	YPO0619	RND	1.0 ± 0.2
*yajR*	*y1017*	NT02YP1187	YPO3169	MFS	1.0 ± 0.1
*arsB*	*y0844*	NT02YP0968	YPO3347	IT	1.0 ± 0.4
*corC*	*y1191*	NT02YP1397	YPO2617	HCC	0.9 ± 0.1
mdtJ*-mdtI	y2242*-y2241	NT02YP2670	YPO2068	SMR	0.8 ± 0.2
*rosA*	*y1087*	NT02YP1268	YPO3093	MFS	0.8 ± 0.1
*ydhC*	*y1948*	NT02YP2306	YPO2389	MFS	0.8 ± 0.2
	*y3186*	NT02YP3792	YPO0798	MFS	0.7 ± 0.1
(*ydhE*)	*y1945*	NT02YP2302	YPO2392	MATE	0.7 ± 0.1
(*ybeQ*)	*y1874*	NT02YP2220	YPO1712	MFS	0.7 ± 0.0
mdlA*-mdlB	y1039*-y1040	NT02YP1213	YPO3145	ABC	0.7 ± 0.0
*ydeF*	*y2653*	NT02YP3168	YPO1515	MFS	0.5 ± 0.1
*emrE, gacE*	*y2000*	NT02YP2368	YPO2333	SMR	0.5 ± 0.0
*mdfA, cmr*	*y4067*	NT02YP4824	YPO4048	MFS	0.2 ± 0.0
(*ydjV*)	*y2272*	NT02YP2703	YPO2040	MFS	nd

^a ^Gene name as annotated for *Y. pestis *strain KIM and/or CO92 or gene names (in parentheses) given herein based on the name of their *E. coli *homologs. Multiple names for the same gene are separated by commas. Genes of multi-component pumps are separated by dashes. The star (*) marks genes from multi-component pump gene clusters whose transcripts were analyzed by qRT-PCR. ^b ^Gene designations in the University of Wisconsin, TIGR, and SANGER *Y. pestis *genome databases. The Wisconsin column shows designations for all the genes in each predicted multi-component pump. TIGR and SANGER columns show only genes targeted in qRT-PCR. ^c ^Pump families assigned based on homology to known (super)family members from other organisms. MFS, major facilitator superfamily; SMR, small multidrug resistance family; ABC, ATP-binding cassette superfamily; RND, resistance-nodulation-cell division superfamily; MATE, multidrug and toxic compound extrusion family; DMT, drug/metabolite transporter superfamily; CDF, cation diffusion facilitator family; ArAE, aromatic acid exporter family; IT, Ion transporter superfamily; HCC, HlyC/CorC family. ^d ^Fold change values (FC) are means of triplicates ± standard errors and are presented in decreasing order. For polycistronic transcripts, qRT-PCR was conducted with primers targeting the first pump component-encoding gene of the operon. These genes are marked with a star. nd, transcript not detected in any of the three *Y. pestis *strains examined (wild-type, *Yp *pGEM-4Z, and *Yp *pGEM-Rob_Yp_).

**Figure 5:**
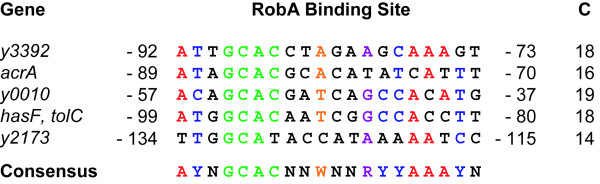
**Potential RobA binding sites in the promoter regions of genes upregulated in *robA*_Yp_-overexpressing *Y. pestis***. The consensus shown is the 20-bp asymmetric *marbox *consensus sequence determined by Martin *et al*., 1999 [37]. R = A or G, Y = C or T, W = A or T, and N = A, T, G, C. Column C is the number of bp's in agreement with the 20-bp consensus sequence. The location of each RobA binding site with respect to the first codon of its cognate gene is indicated by the numbers flanking the putative binding site.

Inspection of the promoter regions upstream of the upregulated genes in *Yp *pGEM-Rob_Yp _revealed the presence of a putative RobA_Ec _binding site in each of these regions (Figure 5). These results suggest that RobA_Yp _may act as a positive regulator for the *y0010, y1050-y1049*, *y2173 *and *y3392-y3393 *systems. This possible regulatory scenario is consistent with the upregulation in the expression levels of these pumps induced by *robA*_Yp _overexpression in *Yp*.

It is likely that the multidrug resistance and solvent tolerance phenotypes induced by *robA*_Yp _overexpression are due, at least in part, to increases in compound extrusion by one or more of the upregulated pumps mentioned above. This idea is supported by the reported observation that the RobA_Ec_-induced multidrug resistance and solvent tolerance in *Ec *is largely dependent on the AcrAB-TolC efflux pump system [25,26].

## Conclusion

The identification of *Yp *genes that, when overexpressed, are capable of reducing antibiotic susceptibility is a useful strategy to expose genes that this pathogen may rely upon to evolve resistance via a vertical modality. In this study, we explored the use of a multicopy suppressor, *Ec *host-based screening approach as a means to identify antibiotic resistance determinant candidates in *Yp*. To seek proof of principle for this approach, we constructed a multicopy plasmid-based, *Yp *genome-wide expression library of nearly 16,000 clones in *Ec *and screened this library for suppressors of the antimicrobial activity of the fluoroquinolone antibiotic OFX. The screen permitted the identification of a gene that, when overexpressed, reduces the susceptibility of *Yp *not only to OFX, but also to other (fluoro)quinolones, tetracyclines and CHL. This gene (*robA*_Yp_) encodes a putative transcriptional regulator, and our results clearly demonstrate that its overexpression in *Yp *and *Ec *confers low-level resistance to multiple antibiotics. Overexpression of *robA*_Yp _also increases organic solvent tolerance in both *Yp *and *Ec *and reduces the susceptibility of *Yp *to zinc.

The molecular mechanism by which overexpression of *robA*_Yp _leads to a reduction in the susceptibility to antibiotics and other compounds remains to be determined. Our results indicate that overexpression of *robA*_Yp _induces a drastic upregulation in the transcript levels of four of the 34 predicted efflux pump gene systems and of *hasF *(*tolC*) in *Yp*. Increased expression of chromosomally encoded efflux pumps is a known cause of multidrug resistance in many bacteria [30]. Thus, it is likely that the reduction in the susceptibility to antibiotics and other compounds induced by *robA*_Yp _overexpression is due, at least in part, to multidrug efflux pump-mediated increases in compound extrusion.

Overall, our findings provide proof of principle for the utilization of an *Ec *host-based suppressor screen to identify antibiotic resistance determinant candidates in *Yp*. This methodology will be useful in the identification of genetic determinants involved in target-dependent and target-independent resistance to antimicrobials with known and unknown mechanisms of action. Identification of such genetic determinants will provide first insights to guide further studies to obtain mechanistic information on novel modes of antimicrobial activity and antimicrobial resistance.

## Methods

### Construction of genomic library

Unless otherwise indicated, all molecular biology and microbiological manipulations were conducted using standard procedures [31] and reagents acquired from New England Biolabs or Sigma-Aldrich. Genomic DNA from the avirulent *Yp *strain KIM6+ [32] was used for the library. This strain lacks the Lcr virulence plasmid [5] and is excluded from the Select Agent Program (please see Availability & requirements for more details). Genomic DNA was prepared using AquaPure™ Genomic DNA Isolation Kit (Bio-Rad Laboratories) and partially digested with *Bsr*FI, which, on average, cleaves the genome of *Yp *every ~760 bp. Independent partial digestions were resolved by agarose gel electrophoresis and the fragment populations in the 4,000-bp to 8,000-bp range were purified using QIAquick Gel Extraction Kit (Qiagen). The fragments were ligated to the multicopy plasmid vector pGEM-4Z (Promega) linearized with *Xma*I and dephosphorylated with calf intestine alkaline phosphatase. Genes inserted into pGEM-4Z can be transcribed from the *gpt-lac *hybrid promoter located at the 5'-end of the cloning site and, potentially, from their native promoters. Ligations were transformed into *Ec *DH5α (Invitrogen) and transformants were selected in Luria-Bertani (LB) agar plates containing AMP (100 μg/ml) and 5-bromo-4-chloro-3-indolyl-β-D-galactopyranoside (40 μg/ml) for blue/white colony screening [31]. White colonies were streaked onto the same medium to verify their white phenotype and 15,648 confirmed white clones were independently grown in AMP-containing LB broth in 96-well plates. After plate incubation for culture growth to early stationary phase, aliquots from each of the 96 cultures of each plate were pooled, cells from each pool were harvested, and plasmids from each pool were purified using QIAprep Spin Miniprep Kit (Qiagen). The cultures in the plates were supplemented with glycerol (25%) and this master library was stored at -70°C. Agarose gel electrophoresis analysis confirmed plasmid population heterogeneity and restriction digestion analysis of plasmids from several clones verified insert diversity (not shown). The library provides a theoretical ~760-fold genome coverage. Our *in silico Bsr*FI restriction analysis of the *Yp *genome revealed the presence of four *Bsr*FI fragments of ≥ 8,000 bp. These fragments, which add up to 39,118 bp, are unlikely to be represented in the library.

### Multicopy suppressor screening

The library was replicated using a 96-pin inoculator (Clonemaster™; Immusine Laboratories, Inc.) to inoculate 96-well plates loaded with fresh culture medium (150 μl/well). After plate incubation for culture growth (9 h, 37°C, 200 rpm), the 15,648 cultures were pooled and the cells of the pool were harvested. The pooled cells were resuspended in fresh medium (1/10 × pool's volume) containing AMP (100 μg/ml) and glycerol (25%), and the suspension was aliquoted (1 ml library stock aliquots) and stored at -70°C. Multicopy suppressor gene-containing clones were screened for by plating a 1/100 dilution of a library stock aliquot on LB agar plates containing AMP (100 μg/ml) and OFX at the MIC (0.35 μg/ml). The OFX MIC was determined by plating *Ec *carrying pGEM-4Z (*Ec *pGEM-4Z) on LB agar plates containing AMP (100 μg/ml) and OFX at increasing concentrations and defined as the concentration for which no colonies were obseved after plate incubation (37°C, 48 h). Clones identified in the screen were streaked on plates containing AMP (100 μg/ml) and OFX (0.35 μg/ml) to confirm their resistant phenotype. The plasmid from each confirmed clone was isolated and transformed into *Ec *and *Yp*. Each transformant was streaked on AMP and OFX-containing plates [LB agar for *Ec *and tryptose blood agar base (TBA; Difco Laboratories) for *Yp*] to ascertain whether the resistance was plasmid mediated. The insert of each plasmid conferring resistance was sequenced using M13 forward and reverse universal primers (Invitrogen). The sequences obtained were used as queries in sequence similarity searches against the *Yp *KIM genome using BLAST (please see Availability & requirements for more details) to determine the genome fragment carried by the plasmid.

### Construction of pGEM-Rob_Yp _and transformation of *Y. pestis*

The fragment encompassing *Yp robA *(herein referred to as *robA*_Yp_) and its promoter region was PCR-amplified from plasmid pGEM-OFXr1 (see results) with primers Robfor1 and Robrev1 (Table 3). The PCR product (1030 bp) was cloned into pCR2.1-TOPO (TOPO TA Cloning Kit, Invitrogen) and the fidelity of the insert was verified by DNA sequencing. The insert was recovered from the pCR2.1-TOPO clone as an *Eco*RI fragment and sub-cloned into the *Eco*RI site of pGEM-4Z using *Ec *DH5α as host. A clone with *robA*_Yp _in the same orientation as the *lacZ *gene of pGEM-4Z was designated pGEM-Rob_Yp_. pGEM-Rob_Yp _and pGEM-4Z were introduced into avirulent *Yp *by electroporation as reported earlier [33] to create strains *Yp *pGEM-Rob_Yp _and *Yp *pGEM-4Z, respectively. *Yp *strains were grown in heart infusion broth (HIB; Difco Laboratories) and on TBA plates without or with antibiotics as appropriate.

**Table 3:** Oligonucleotides used in this study

**Name^a^**	**Sequence**
Robfor1	5'-TCTAGACGCTTTTTAACACACTGTACCAGT-3'
Robrev1	5'-GAATTCATTTAGATATGCCAGCACTTGATGA-3'
16sRNAF	5'-ATGACCAGCCACACTGGAACTGA-3'
16sRNAR	5'-TGACTTAACAAACCGCCTGCGT-3'
y3392F	5'-AGCGGCACCTTGGTCAATATTGT-3'
y3392R	5'-CAATTTGGTTATCCACCGATTCA-3'
y1050F	5'-GCTTATGACAGTGCAAAAGGTGA-3'
y1050R	5'-GATTAATGCGTGCAGACTCCAGT-3'
y0702F	5'-TATACCCAAGTGCGGGCACCCAT-3'
y0702R	5'-CATTCGCTACTGTGTCATTGCCT-3'
y3402F	5'-TCGATGCCACTGAATAGCGATCT-3'
y3402R	5'-ATCTGGTGAACGCAATAACGAGT-3'
y1439F	5'-CAGCCATCAAGAGGCTGCCCCAA-3'
y1439R	5'-ACCAAAGGCATCGACGCTGCCGA-3'
y1087F	5'-GGTGCTATCAGCGTATCTCACCT-3'
y1087R	5'-CCATACCGATGGGTAATGAGTAT-3'
y0060F	5'-GCAACCTGCTGATGAAGAACATA-3'
y0060R	5'-CACGAATCGCCTGACTGTGTGTT-3'
y0844F	5'-CGTTGCTAAACCGACTGGGTGAA-3'
y0844R	5'-TTGCGACAAAACATGCAGCCACA-3'
y0922F	5'-CGGCAGTGTGGTCAGTGTTCATT-3'
y0922R	5'-CACCCGACGCTTCAGGTCATTTT-3'
y1191F	5'-TCCTTAACCAGCTCTTCCACGGT-3'
y1191R	5'-CTTTATCTTCGCTGATCACCGGA-3'
y1386F	5'-GTGGTCAGGACACTAGCCATGGT-3'
y1386R	5'-CCTGCGTCAGTTGGACTTCGTAA-3'
y1481F	5'-AAAGCACAGAAGCAGGTGACTGT-3'
y1481R	5'-TTGTTGCTGACGCTGGTAGGTGA-3'
y1874F	5'-TTACCGACTATCGCACGTGACCT-3'
y1874R	5'-TTGTAGCACACGGGCAAACGTCA-3'
y1945F	5'-CCTGATGATGAGATGGTACGTAA-3'
y1945R	5'-TGAACTGAAGTTGAGGGCAATCT-3'
y2242F	5'-TTTAGTGACACTGATTGGTGGGA-3'
y2242R	5'-CATGATGCTCACCTGACTCAACA-3'
y3180F	5'-GGTAATGATGGTGGCTAATGGTT-3'
y3180R	5'-CAACCGAGCCAAGTAAGATCGCA-3'
y3558F	5'-GCCATTGATCCTGTTATCGGCTA-3'
y3558R	5'-ATAGGGAACAGATGAATGCCACA-3'
y4041F	5'-TTACGACCACAACGGTAGATGAA-3'
y4041R	5'-CATTGGTGCGGCAAGGTTCATAT-3'
y4067F	5'-GATATGATCCAGCCAGGTATGCT-3'
y4067R	5'-GCACCGATAAAGCACAAGCCAAT-3'
y0010F	5'-TCGCCGAAAGCCTTAACCGTTCT-3'
y0010R	5'-CCGAGAACGCACTAAGAAAGCCA-3'
y0178F	5'-TTTACCGCAGACGTGGTCGCTAT-3'
y0178R	5'-CCTACTGGACTCCCGTTGCTTCT-3'
y0613F	5'-GGGCTATTGGCCTGAAGTATTCT-3'
y0613R	5'-AAGCTTAGTATCCGCGCCAGACT-3'
y1017F	5'-AAATGACTCCGCTAGAGCTTCGA-3'
y1017R	5'-GGTTTACGACCGATACGATCAGA-3'
y1039F	5'-GCCGTGAATGGCACCGTTATGTA-3'
y1039R	5'-AGTTTGCCGACTCAGCTGACGAT-3'
y1948F	5'-CTGCCTGTAGTGCTGGCTTCTTT-3'
y1948R	5'-AGTACAGGGCAATCATGCTGCCA-3'
y2000F	5'-TGGCGATTATTGCCGAAGTGGTT-3'
y2000R	5'-AAAGGTGGCAGCGATTGAGACCA-3'
y2108F	5'-GGTTGTATATCAGCGGTAGTTCT-3'
y2108R	5'-TTGCACGAAAGTGTTTAGACGCT-3'
y2173F	5'-ACAAGGTGTCTCGGTATGCTGCA-3'
y2173R	5'-ATAATGCCAGGAACCAGAACGCT-3'
y2272F	5'-TTGGTATCGCAAGCTCGAAGCTT-3'
y2272R	5'-TCGCATTAGCATCCCGGTGACAA-3'
y2653F	5'-ATGACCGTCAATGCGACCATCGT-3'
y2653R	5'-AATGGCCATTGCCAGCATCCATA-3'
y2814F	5'-TCTGGACCAGGCAGTAACCGATT-3'
y2814R	5'-TACTCATATCGGCCAGGGTCAGA-3'
y2916F	5'-CCTTGGGTTGTTGTCGATGCTGA-3'
y2916R	5'-ACATGCCATACCTGCAAGCGCAA-3'
y3186F	5'-GTCAGTTGGACGTTACTGCTAAT-3'
y3186R	5'-CTTTCTTGCCATAAGCGACGACA-3'
y3760F	5'-TCTGGATATTCGCCGTGCAGAGA-3'
y3760R	5'-CGTGGTAAACAGACGCTCTGGAA-3'
y3516F	5'-TGCAACGACTAACCTGTATCAGT-3'
y3516R	5'-TTTGGCGAGTAGTATTCTCTGGT-3'

^a ^Primers used for qRT-PCR were named according to the University of Wisconsin gene designations for the *Y. pestis *KIM genome, except for 16sRNAF-R (used to amplify 16s rRNA).

### MIC_99 _and IC_50 _determinations

Dose-response experiments were done in triplicate and using 96 well plate-based microdilution assays as reported [12,14]. Briefly, wells contained 200 μl of broth (LB for *Ec*, HIB for *Yp*) inoculated with 10^4 ^cfu/ml and supplemented with AMP (100 μg/ml) and a second antimicrobial compound at the concentration indicated below. Antimicrobial compounds were added from stock solutions in water, ethanol, or DMSO. Control cultures lacking the antimicrobial compounds contained water (2%), ethanol (1%), or DMSO (0.5%). After incubation (37°C, 200 rpm, 24 h for *Yp *and 16 h for *Ec*), growth was measured as optical density (*A*_620_) using a Spectra Max Plus spectrophotometer plate reader (Molecular Dynamics). IC_50 _values were calculated from sigmoidal curves fitted to triplicate sets of dose-response data using KaleidaGraph (Synergy Software). MIC_99 _values were calculated as the lowest concentration tested that inhibited growth by ≥ 99%. The range of concentrations tested were: OFX (Sigma), 2.5-0.001 μg/ml; KAN (Shelton Scientific), 50-0.024 μg/ml; CHL (Calbiochem), 10-0.005 μg/ml for *Yp *and 25-0.012 μg/ml for *Ec*; TET (Sigma), 40-0.020 μg/ml; APR (Sigma), 50-0.024 μg/ml; NAL (Sigma), 25-0.012 μg/ml for *Yp *and 400-0.195 μg/ml for *Ec*; STR (Sigma), 50-0.024 μg/ml for *Yp *and 100-0.049 μg/ml for *Ec*; GEN (EM Science), 25-0.012 μg/ml for *Yp *and 50-0.024 μg/ml for *Ec*; DOX (Sigma), 10-0.005 μg/ml; CIP (Fluka), 0.75-0.0004 μg/ml for *Yp *and 1.25-0.0006 μg/ml for *Ec*; LVX (Fluka), 0.75-0.0004 μg/ml for *Yp *and 1.25-0.0006 μg/ml for *Ec*; plumbagin (Sigma), 50-0.024 μg/ml for *Yp *and 200-0.098 μg/ml for *Ec*; menadione (Sigma), 50-0.024 μg/ml for *Yp *and 400-0.195 μg/ml for *Ec*; paraquat (Sigma), 200-0.098 μg/ml for *Yp *and 400-0.195 μg/ml for *Ec*; CoCl_2 _(Sigma), 1-0.0005 mg/ml; CuSO_4 _(Sigma), 2-0.001 mg/ml for *Yp *and 4-0.002 mg/ml for *Ec*; ZnCl_2 _(Sigma), 1-0.0005 mg/ml.

### Organic solvent tolerance assay

The test for solvent tolerance was conducted essentially as reported previously [21]. Overnight cultures of *Ec *and *Yp *strains grown in LB broth and HIB, respectively, were inoculated (1%) into fresh media and allowed to grow to *A*_620 _= 0.4. Then, 5 μl of each culture were spotted on solid medium (LB agar for *Ec*, TBA for *Yp*) with 100 μg/ml AMP for transformants carrying pGEM plasmids or without antibiotic for other strains. The surface of the medium was then overlaid with the organic solvent (7 ml) to a thickness of ~3 mm. The plates were sealed and incubated for 24 h for *Ec *strains and 48 h for *Yp *strains before naked-eye examination for bacterial growth.

### Time-kill experiments

*Yp* pGEM-Rob_Yp _and *Yp *pGEM-4Z (control) were treated with STR, GEN, OFX, or CIP at 5 × MIC_99_. The MIC_99 _values were those determined using *Yp *pGEM-4Z in the dose-response experiments above (5 × MIC_99 _values: STR, 15.6 μg/ml; GEN, 5.2 μg/ml; OFX, 0.17 μg/ml; CIP, 0.12 μg/ml). For each antibiotic tested, three tubes with 10 ml of preheated (37°C) HIB containing AMP (100 μg/ml) were inoculated with 10 μl of an overnight culture of the corresponding *Yp *strain and incubated at 37°C with shaking at 200 rpm for 2 h. After incubation, a sample of each culture was taken and cfu/ml were determined by plating serial dilutions on TBA plates containing AMP (100 μg/ml) and enumerating colonies after plate incubation. Immediately after culture sampling, the test antibiotic was added (from stock solutions in water for GEN and STR or stock solutions in DMSO for OFX and CIP) and the cultures were returned to incubation (37°C, 200 rpm). Samples from these cultures were then taken at time points 0 (immediately after antibiotic addition), 30, 60, 90, 120, 180, 240, 300, 360, and 420 min for cfu/ml determination as above. Triplicate control cultures where water or DMSO was added in place of the antibiotic solution were included in the experiments and treated and analyzed in the same way as the antibiotic-treated cultures. The time-kill data were plotted using Kaleidagraph (Synergy software).

### Isolation of total RNA and qRT-PCR

*Yp *and *Ec *were cultured in HIB and LB broth, respectively. AMP (100 μg/ml) was added to the medium for strains carrying pGEM-Rob_Yp _or pGEM-4Z. Cultures were incubated (37°C, 200 rpm) until they reached *A*_620 _of ~0.5 before RNA was isolated using the RiboPure-Bacteria Kit (Ambion) according to the manufacturer's instructions. RNA was isolated from triplicate cultures and treated with DNase I (Ambion) (4 units, 37°C, 30 min) in DNase I Buffer (Ambion). After the treatment, DNase I was inactivated by adding DNase Inactivation Reagent (Ambion) at 20% of the final volume of RNA treated. The inactivation was allowed to proceed at room temperature for 2 min. The RNA sample was then centrifuged at maximum speed in a microcentrifuge for 1 min to pellet the inactivation reagent. The RNA was then transferred to a new RNase-free microcentrifuge tube. cDNA was prepared from each RNA sample using TaqMan^® ^Reverse Transcription Reagents Kit (Applied Biosystems) according to the manufacturer's instructions. Each cDNA sample was analyzed in triplicate by qRT-PCR using SYBR^® ^Green Master Mix (Applied Biosystems) according to the manufacturer's instructions. cDNA was kept undiluted for qRT-PCR analysis of *robA *cDNA and diluted 1:5 for analysis of other cDNAs. qRT-PCR and target sequence relative quantification were carried out using a 384-multiwell platform with an ABI-PRISM 7900 HT Sequence Detection System (Applied Biosystems) as described previously [34,35]. The thermocycling program included 1 cycle of 95°C for 5 min followed by 40 cycles of 95°C for 30 sec, 55°C for 30 sec, and 72°C for 1 min. Relative quantification was conducted using the standard equation 2^-ΔΔCT ^{*i.e*. 2^- [(CT of target cDNA in sample1-CT of 16S rRNA cDNA in sample 1)-(CT of target cDNA in sample 2-CT of 16S rRNA cDNA in sample 2)]^} [36]. The equation expresses n-fold difference of the target cDNA in the sample from the strain carrying pGEM-Rob_Yp _(sample 1) relative to the target cDNA in the sample from the strain carrying pGEM-4Z (sample 2, control) with normalization to an endogenous control (16S rRNA cDNA). The cycle threshold (CT) values utilized in the equation were the average of three independent cultures of the same strain, each analyzed in triplicate by qRT-PCR.

### Identification of putative drug efflux pumps and RobA_Yp _binding sites

Searches for efflux pumps were conducted in the sequenced genomes of *Yp *KIM and and *Yp *CO92 (please see Availability & requirements for more details). The search included the following strategies. First, the tables of functional classes in the genome websites were examined for annotated pumps. Second, the navigator function in the Artemis genome viewer software (please see Availability & requirements for more details) was used to search for the terms multidrug, efflux, transport, translocase, pump, and drug resistance as qualifiers in the annotated genome sequences. Third, the names of annotated *Ec *multidrug efflux pumps were used as search keywords using the navigator function in Artemis to find potential pumps not yet identified with the other strategies. Fourth, all putative pumps identified in *Yp *KIM were used as queries in BLASTP-based searches against the *Yp *CO92 genome and *vice versa*. Fifth, annotated *Ec *multidrug efflux pumps were used as queries in BLASTP-based searches against the *Yp *KIM and *Yp *CO92 genomes. Potential RobA_Ec _binding sites were searched for using the navigator function in Artemis and the naked eye.

## Abbreviations

AMP: ampicillin; APR: apramycin; CHL: chloramphenicol; CIP: ciprofloxacin; CT: cycle threshold; DOX: doxycycline; *Ec*: *E. coli*; GEN: gentamicin; HIB: heart infusion broth; KAN: kanamycin; LB: Luria-Bertani; LVX: levofloxacin; MDR: multidrug-resistant; MFS: major facilitator superfamily; NAL: nalidixic acid; OFX: ofloxacin; RND: resistance-nodulation-cell division; STR: streptomycin; TBA: tryptose blood agar base; TET: tetracycline; *Yp*: *Y. pestis*.

## Availability & requirements

CD-Search: 

Select Agent Program: 

BLAST: 

Yersinia pestis KIM Genome Page: 

University of Wisconsin *E. Coli* Genome Project: 

Wellcome Trust, Sanger Institute, Yersinia pestis data: 

Artemis genome viewer software: 

## Authors' contributions

LENQ, KLS, and JAF conceived and designed the experiments. KLS, JAF, SMR, and FVF constructed the expression library. KLS and SMR screened the library. KLS carried out the gene expression analysis and bioinformatic-guided identification of efflux pump genes. KLS and RLM constructed all plasmids and strains and conducted the strain characterization experiments. All authors contributed to the preparation of the manuscript. LENQ and KLS wrote the final version of the manuscript. All authors read and approved the final version of the manuscript. LENQ directed and oversaw the project.
